# Cytomegaloviruses reorganize endomembrane system to intersect endosomal and amphisome-like egress pathway

**DOI:** 10.3389/fcell.2023.1328751

**Published:** 2023-12-19

**Authors:** Pero Lučin, Hana Mahmutefendić Lučin, Gordana Blagojević Zagorac

**Affiliations:** ^1^ Department of Physiology, Immunology and Pathophysiology, Faculty of Medicine, University of Rijeka, Rijeka, Croatia; ^2^ University North, University Center Varaždin, Varaždin, Croatia

**Keywords:** betaherpesviruses, cytomegalovirus, assembly compartment, secondary envelopment, virion egress, multiviral bodies

## 1 Introduction

Cytomegaloviruses (CMV) cause productive, persistent or latent infections in their hosts. Productive infection requires the establishment of a complete manufacturing chain in the nucleus and cytosol for the successful production and release of infectious virions. This establishment is associated with overproduction of virus-encoded proteins and extensive reorganization of host cell pathways. The cytoplasmic part of the manufacturing chain involves a complex reorganization of the membrane system into a large structure known as the assembly compartment (AC), which adjusts the membrane composition to concentrate all essential components for the final stage of virion production known as the secondary envelopment and provides the exit pathway for the release of assembled virions from the cell (Wofford et al., 2020). These crucial steps in the CMV life cycle (Supplementary Figure S1) are still poorly understood and need to be regularly reconciled with advances in herpesvirus biology and membrane trafficking.

## 2 Cytoplasmic assembly compartment

The extensive reorganization of the membrane system has been investigated in several electron microscopy (EM) (Maninger et al., 2011; Schauflinger et al., 2013; Read et al., 2019; Taisne et al., 2019; Momtaz et al., 2021; Flomm et al., 2022; Wedemann et al., 2022) and immunofluorescence-based studies (Supplementary Table S1). This reorganization involves displacement the Golgi stacks into a ring-like configuration (outer AC) which surrounds the expanded membranous elements (inner AC), mainly the intermediates of the interface between early endosomes, the endosomal recycling compartment, and the trans-Golgi network (EE-ERC-TGN) (Das et al., 2007; Lučin et al., 2020). The overall structure relocates late endosomes (LE) and the endoplasmic reticulum (ER) to the cell periphery. Membrane expansion in the inner AC is associated with extensive tubulation and sustained recruitment of tubulation machinery. In addition, the AC is rich in vacuolized membrane structures that are often loaded with enveloped virions.

An important goal of membrane reorganization is to create a suitable environment for membrane envelopment of the nucleocapsids and to pave the exit pathway for the enveloped virions. These changes, initiated in the early phase of infection and completed after viral DNA replication, include redistribution and over-recruitment of proteins that regulate membrane trafficking (Beltran et al., 2016; Cook and Cristea, 2019; Lučin et al., 2020), alteration of lipid composition (Liu et al., 2011), retention of membrane trafficking and inhibition of endosomal recycling (Tomaš et al., 2010; Hook et al., 2014; Karleuša et al., 2018; Maschkowitz et al., 2018), and even establishment of heterotypic membranous organelles within the endosomal system in the post-sorting phase of endosomes (Cepeda et al., 2010; Zeltzer et al., 2018). However, little is known about the biogenesis and membrane composition of thousands of membranous units that make up the AC.

At least 14 virus-encoded tegument proteins and 11 glycoproteins are embedded in virions during the secondary envelopment (Varnum et al., 2004), and their concentration at the envelopment organelle is required. Studies focusing on the trafficking of highly conserved glycoproteins (including the gB, gH/gL/gO and gM/gN complexes) (Tugizov et al., 1999; Jarvis et al., 2002; Crump et al., 2003; Krzyzaniak et al., 2009; Maschkowitz et al., 2018) revealed their different maturation pathways. They concentrate in the trans-Golgi and circulate via different routes through the PM, endosomes and TGN. Several studies suggest that the clathrin-dependent endocytic uptake of viral glycoproteins, their delivery into the endosomal system and retrieval at the recycling membranes may be essential for the process of secondary envelopment (Krzyzaniak et al., 2009; Kropff et al., 2010; Wu et al., 2020). The identification of endosomal host cell cargo proteins (i.e., MHC class I proteins and Lamp1), several Rab GTPases (i.e., Rabs 12, 18, 23, and 32), cargo sorting proteins (i.e., sorting nexins 2 and 3), SNARE proteins (i.e., STX12, VAMP2, and VAMP3), and lipid-modifying proteins (i.e., PIK3C2A) as host cell membrane signatures that remain in virions (Mahmutefendić Lučin et al., 2022), support the idea that the distant stage of EE maturation in one of the endosomal recycling pathways may provide the retrieval machinery required for retention of viral glycoproteins. On the other hand, little is known about the concentration of tegument proteins, but they may be retained at the envelopment membrane by direct interaction with the retrieval machinery or by complex formation with retrieved glycoproteins (Seo and Britt, 2006; Wu et al., 2020).

## 3 Secondary envelopment

The EM studies agree that secondary envelopment occurs within the AC. Enveloped capsids have been observed as single capsids, but the hallmark of CMV infection is their accumulation in large vesicles, often referred to as MVBs or, better, multiviral bodies (MViBs), as recently proposed (Wedemann et al., 2022). Some CMVs, such as murine CMV in fibroblasts, are enveloped as large multicapsid virions (Maninger et al., 2011).

The identity of the envelopment membranes and the mechanism of CMV envelopment are still unknown. Studies of the trafficking of viral envelope proteins point to the trans-Golgi (Jarvis et al., 2002; Homman-Loudiyi et al., 2003; Cepeda et al., 2010), EEs (Tooze et al., 1993; Radsak et al., 1996; Fraile-Ramos et al., 2002), and the ERC (Krzyzaniak et al., 2009; Lučin et al., 2018; Lučin et al., 2020). There are also several lines of evidence that CMV maturation occurs within the membranous system along endocytic flux (Archer et al., 2017; Hasan et al., 2018; Štimac et al., 2021). Knockdown of host cell factors regulating membrane flux suggests that EE maturation is essential for virion envelopment and release (Sharon-Friling and Shenk, 2014; Pavelin et al., 2017; Turner et al., 2020).

Secondary envelopment is initiated by the contact of tegumented capsids with the membranous compartment, causing the membrane to deform and wrap around either as a double membrane or as budding into a larger compartment (Close et al., 2018; Wofford et al., 2020). Advanced EM has shown that CMV envelopment occurs at complex tubular structures that provide sufficient membranes to wrap around the capsids (Maninger et al., 2011; Schauflinger et al., 2013). These events are evenly distributed across the AC, and no preferred site for envelopment has been identified. At a later stage of infection, capsids were observed in large vacuoles at the periphery of the AC, leading to the proposal that envelopment occurs by budding in MVBs (Fraile-Ramos et al., 2002; Flomm et al., 2022; Wedemann et al., 2022). However, suppression of components of the endosomal sorting complex required for transport (ESCRT), which is essential for reverse budding into MVBs and MVB maturation toward the degradation pathway, did not affect CMV maturation (Fraile-Ramos et al., 2007), but further studies showed that the ESCRT-III machinery is involved in the final steps of HCMV replication (Tandon et al., 2009), likely in virion release and virus spread (Streck et al., 2018). A recent study demonstrates that ESCRT-III component CHMPC may be either required for scission of a pool of wrapping membranes derived from recycling endosomes used for envelopment of HSV1 or closure of membranes around virions analogous to the sealing of phagophore in autophagy (Russell et al., 2021). Proteomic and lipidomic analysis of extracellular virions identified several host cell signatures within virions that suggests envelopment at membranes derived from recycling EEs and did not reveal any signatures that might indicate envelopment at MVBs (Mahmutefendić Lučin et al., 2022). Thus, there is little evidence that MVBs can be used for envelopment.

## 4 Egress route

Alpha-herpesviruses exit cells as single particles via secretory vesicles and exosomes (Bergeman et al., 2023), whereas beta- (Bughio et al., 2015; Momtaz et al., 2021) and gamma-herpesviruses (Peng et al., 2010) appear to collect in large MVB-like membranous organelles before exiting cells. The exit of CMVs has been recently described as intermittent bulk release of many particles that attach to PM prior release into the extracellular fluid (Flomm et al., 2022; Wedemann et al., 2022). Virion-containing MVBs have been observed in several cell types and appear to represent a form of the non-conventional secretion pathway (Turner et al., 2020; Flomm et al., 2022). In fibroblasts, they are likely generated from classical LEs, whereas in endothelial cells they originate from a nonclassical Golgi-mediated exit pathway (Momtaz et al., 2021). The pathway by which enveloped virions are collected and the biogenesis of multivesicular organelles associated with CMV infection remain poorly understood. It is also not known whether CMVs can also envelop into single vesicles and be released as single virions by direct fusion with the PM, as suggested by (Turner and Mathias, 2022).

## 5 Discussion and further directions

**FIGURE 1:**
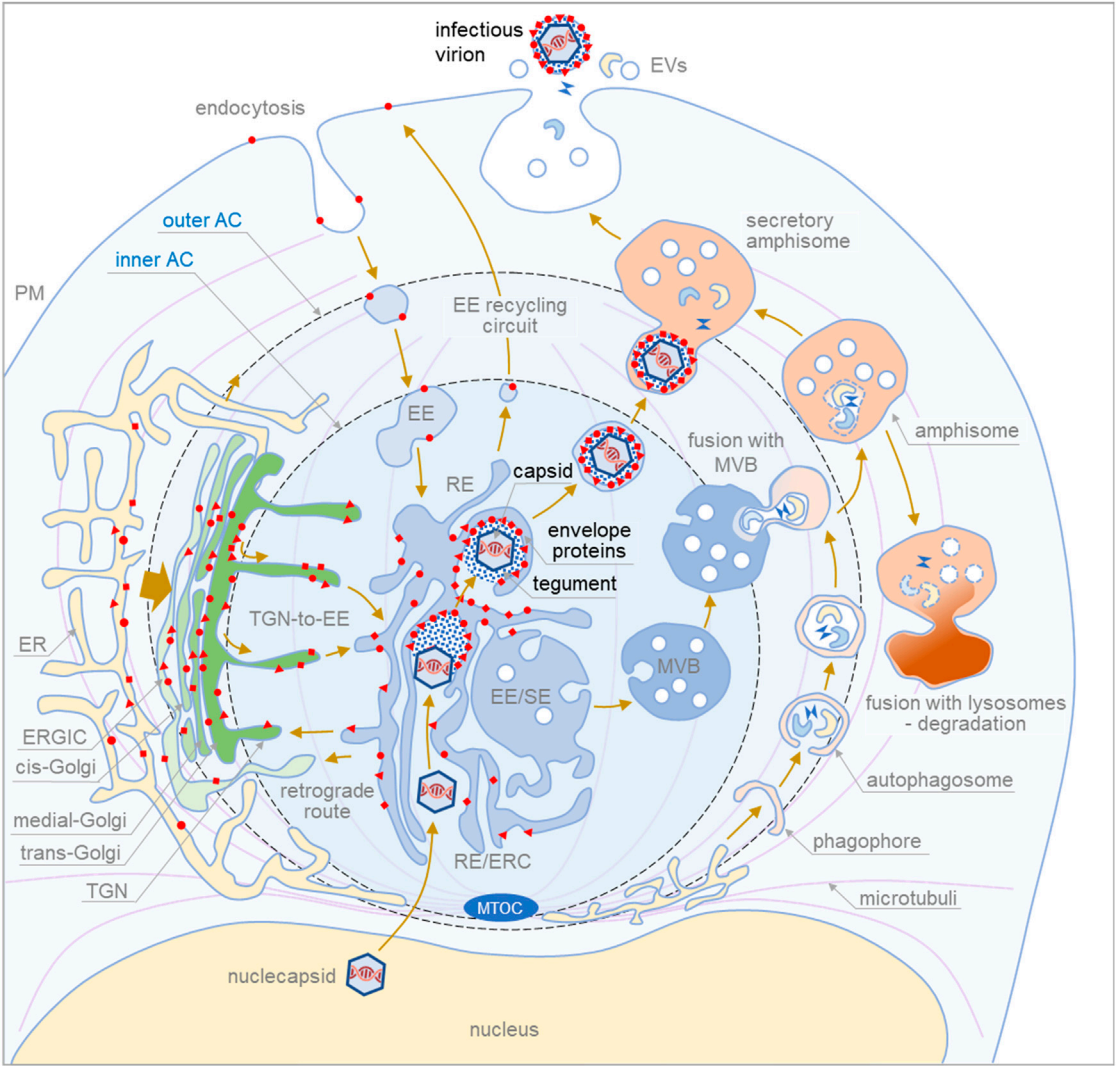
Proposed sequence of cytoplasmic events during final envelopment and exit of cytomegaloviruse. The membrane system of the infected cell is reorganized into a large structure called the assembly compartment (AC). The Golgi stacks and trans-Golgi network (TGN) are relocated to the periphery of the AC in the form of a ring called the outer AC. Late endosomes (LE) and the endoplasmic reticulum (ER) remain outside the ring. In contrast, early endosomes (EE), recycling endosomes (RE)/endosomal recycling compartment (ERC) and TGN remain inside the ring (inner AC). Endocytic flux is directed to the inner AC where EE undergoes maturation. CMV infection halts downstream maturation of EEs and expands membrane elements at the EE-RE/ERC-TGN interface, which accumulate in the inner AC. EEs that mature into multivesicular bodies (MVBs) move outside the AC to join LE maturation pathways. Autophagic processes within the AC and fusion with MVBs to form amphisomes occur at the edges of the AC. Degradative amphisomes fuse with lysosomes, and secretory amphisomes dispose of cellular components outside the cell by fusing with the membrane. During CMV infection, virion components are formed in the late phase of infection. Viral envelope proteins are synthesized in the ER, modified, accumulated in the trans-Golgi/TGN (outer AC), and transported to the membrane system of infected cells. Membrane flux transports the envelope proteins to the membranes of the inner AC, and the cargo sorting and cargo retrieval machinery of the EEs retrieves the coat proteins at membranes with a composition suitable for secondary envelopment. This composition, which includes lipid-modifying proteins and curvature-promoting lipids and proteins, is achieved in the final steps of EE maturation in recycling pathways at modified RE-like membranes extending within the AC. Tegument proteins accumulate in the cytosol of the inner AC and form biomolecular condensates on membranes that concentrate envelope proteins. Viral capsids released from the nucleus embed into the tegument matrix and initiate the envelopment of the envelope protein-rich membrane, which wraps around the material containing the capsids, tegument proteins, and host cell proteins (signatures) to generate enveloped virions. The process of envelopment is similar to the growth of phagophores. Enveloped virions within membrane organelles migrate and fuse with secretory amphisomes, which collect multiple virions and form Multi Viral Bodied (MViBs). MViBs migrate to the cell periphery and release many virions simultaneously, which is known as intermittent bulk release.

The available information is insufficient to reconstruct the secondary envelopment and exit pathways of CMVs and should be reconciled with the growing knowledge of multiple sorting pathways at the EE-ERC-TGN interface, heterogeneity and redundancy of secretory pathways, and their overlap with secretory autophagy pathways. With this in mind, we propose the following scheme of BHV maturation (Figure 1) and highlight critical steps that should be clarified to fully understand BHV envelopment and egress.

To form the secondary envelopment site, CMVs should adapt protein and lipid composition that can concentrate envelope and tegument proteins and engulf large tegumented virions (multicapsids in the case of MCMV) into membrane organelle. For concentrating envelope proteins, CMVs can co-opt cargo sorting and retrieval machinery (i.e., sorting nexins, retrieving complexes), a function mostly associated with EEs. Envelopment can occur after the retrieval at membranes that can grow and bend around tegumented capsids, a property that characterize endosomal tubulation. Indeed, high membrane tubulation is a hallmark within the AC (Lučin et al., 2018; Lučin et al., 2020) and may be required for autophagosome-like membrane wrapping around tegumented capsids. However, no single biochemical remnant can be found in released virions as a signature of the conventional autophagy pathway, and on the other hand, many signatures found suggest envelopment at RE-derived membranes (Mahmutefendić Lučin et al., 2022). What triggers membrane wrapping around tegumented capsids remains unknown, and one mechanism could be the formation of tegument-initiated biomolecular condensates at the site of glycoprotein and nucleocapsid concentration, as has been proposed for herpes simplex virus (Metrick et al., 2020). In addition to protein machinery, lipid composition should also facilitate expansion and wrapping of the envelopment membranes. In the inner AC, membranes are rich in phosphatidylserine (PS) (Lučin et al., 2020), a cylindrical phospholipid required for elongation and PS should be replaced by conical phospholipids such as phosphatidylethanolamine (PE), which facilitates negative curvatures (McMahon and Boucrot, 2015). Indeed, proteomic (Turner et al., 2020) and lipidomic (Liu et al., 2011) analyses of virions revealed the absence of phosphoinositides, PS and PS-binding proteins, and enrichment of PE and PE-binding proteins.

Although envelopment on MVB-like structures has been proposed (Flomm et al., 2022), remnants of the machinery that drives the EE to LE maturation, sorts proteins into intralumenal vesicles, and performs the reverse budding process (i.e., ESCRT) on MVBs have been found in virions (Mahmutefendić Lučin et al., 2022). Most LE-derived MVB structures are dislocated outside the AC (Supplementary Table S1; (Lučin et al., 2020), and most envelopment events occur within the AC (Schauflinger et al., 2013). Nevertheless, envelopment on large organelles cannot be ruled out if forces other than the reverse budding machinery are responsible for envelopment. The ESCRT-III may contributes to the final stages Click or tap here to enter text and may be essential for membrane sealing in the wrapping processes, as in autophagosome maturation (Jiang et al., 2021).

To find a way out of the cell, CMV-containing vesicles should fuse with large transport vehicles that travel to the cell surface, as traveling within large MViBs appears to be the main exit route (Flomm et al., 2022). CMV-containing vesicles can fuse either with the continuous stream of large multivesicular organelles known as amphisomes that transport secretory autophagic contents out of the cell (Ganesan and Cai, 2021), or with degradative amphisomes and divert their route to the PM by delivering RE-derived factors, or even with LE- or TGN-derived vessels that form lysosome-related organelles (LRO) (Delevoye et al., 2019). It appears that the large vessels that transport CMV for release are heterogeneous: In fibroblasts, they are more associated with the LE-derived secretory pathway, whereas in endothelial cells, they correspond more to the autophagic pathway (Momtaz et al., 2021). In addition, virion-loaded organelles can undergo a maturation process similar to that described for LRO and be released by a biphasically evoked exocytic mechanism controlled by the GTPases Rab3a/Rab15/Rab35, as described for LRO (Biesemann et al., 2017), or by exocyst and Rab11b as for lysosome-associated exocytosis (Escrevente et al., 2021).

In conclusion, to understand the final steps of CMV production, it would be important to identify the envelopment organelle and the organelle that transports virions out of the cell. The roadmap requires identification of small GTPases (Rab proteins or others), tethering complexes, and SNARE proteins in both organelles. To achieve this, fluorescently labeled capsids would need to be observed throughout the infected cell over a long period of time, using live cell imaging technology with high resolution and very low phototoxicity. This appears to be a difficult task for CMV, and there are only a few reports (Sampaio et al., 2005; Bosse et al., 2012) on capsid-labeled virions. An additional obstacle for more in-depth studies is the heterogeneity of cells, which allows the establishment of the final steps of the assembly chain only in a limited number of cells. Therefore, cytoplasmic MViB transport appears to be a relatively rare event, and single-cell analyzes with labeled capsids are essential for further progress in the field of secondary envelopment and egress of CMVs.

Unlike alpha-herpesviruses and other viruses, CMV extensively remodel the cell’s membrane system to form unique compartments for their envelopment, egress, and possibly signaling (Henaff et al., 2012). Understanding these changes may therefore not only contribute to a better understanding of CMV biology, but also to elucidating the physiology of the membrane system. Accordingly, a detailed understanding of these processes would pave the way for further development of host-directed antiviral therapy, an emerging concept to develop broad-spectrum drugs to counteract the evolution of viral resistance (Kumar et al., 2020).
