# Supplementary material for: Cytomegaloviruses reorganize endomembrane system to intersect endosomal and amphisome-like egress pathway
Source: Front Cell Dev Biol. 2023 Dec 19;11:1328751. doi: 10.3389/fcell.2023.1328751 (PMC10766366; doi:10.3389/fcell.2023.1328751)
Supplement: Supplementary file 1 [file DataSheet1.docx]

Supplementary Material

# Supplementary Figure 1

#
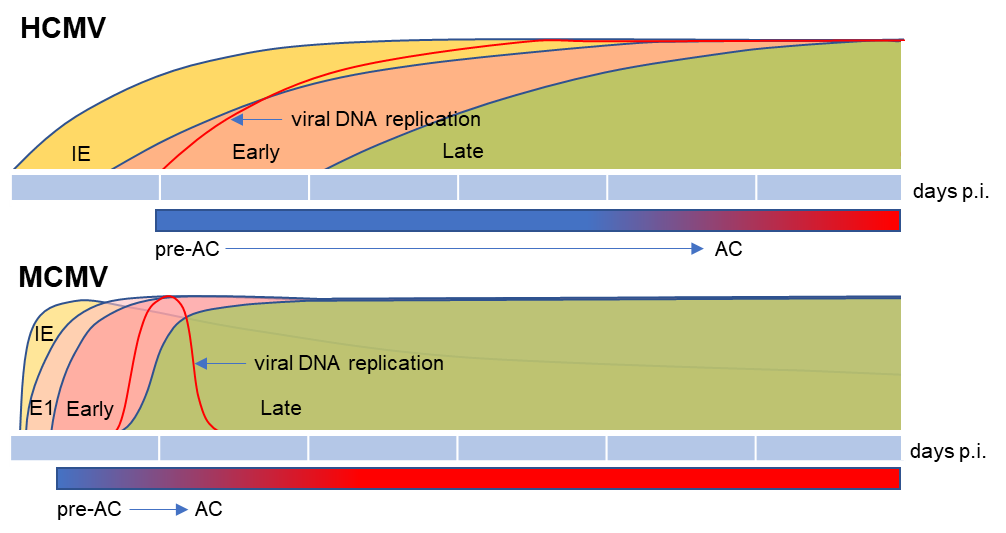


*Figure S1*. **Timeline of viral gene expression and DNA replication of HCMV and MCMV**. (HCMV) In human fibroblasts, immediate early (IE) gene products (yellow) and early gene products (pink) are expressed within 24 hpi with a gradual increase over time. Viral DNA replication (red line) is initiated by 24 hpi and gradually increases until it reaches a plateau at 72-96 hpi. Late genes are expressed after 48 hpi (green). Adapted from (Sanchez & Britt, 2022). Reorganization of the membrane system representing the pre-AC begins around 24 hpi and ACs appear around 108 hpi in some infected cells and are fully developed by 144 hpi in most infected cells (Das et al., 2007). (MCMV) In murine fibroblasts, IE gene products are expressed 1-2 hpi, followed by the first set of early (E1) gene products 2-3 hpi and most early genes products from 4-5 hpi (Lacaze et al., 2011). Viral DNA synthesis starts 15-16 hpi and most of the viral DNA is replicated 16-28 hpi (Mahmutefendić Lučin et al., 2023; Marcinowski et al., 2012). The products of late genes are expressed after 16 hpi (Lacaze et al., 2011; Marcinowski et al., 2012). Reorganization of the membrane system and establishment of the pre-AC is initiated 5-6 hpi, is present in most infected cells 8-10 hpi and loaded with viral structural proteins 16-20 hpi (Karleuša et al., 2018, 2020; Lučin et al., 2020; Marcelić et al., 2021; Štimac et al., 2021; Tomaš et al., 2010). ACs are observed 20-24 hpi in some infected cells and after 48 hpi in half of infected cells (Lučin et al., 2020).

# Table S1. Structure of HCMV-induced assembly compartment

| **Marker** | **Localization** | | | | **Reference** |  | **Marker** | **Localization** | | | | **Reference** |
| --- | --- | --- | --- | --- | --- | --- | --- | --- | --- | --- | --- | --- |
|  | Inner AC | Outer AC | Out | Virions |  |  |  | Inner AC | Outer AC | Out | Virions |  |
| **RAP** |  |  |  |  | Sanchez et al., 2000 |  | **SNAP23** |  |  |  |  | Liu et al., 2011 |
| **ERGIC53** |  |  |  |  |  |  | **TGN46** |  |  |  |  | Hook et al., 2014 |
| **GM130** |  |  |  |  |  |  | **GM130** |  |  |  |  |  |
| **MAN II** |  |  |  |  |  |  | **GOLGIN 245** |  |  |  |  |  |
| **TGN46** |  |  |  |  |  |  | **TfR** |  |  |  |  |  |
| **LAMP1** |  |  |  |  |  |  | **EEA1** |  |  |  |  |  |
| **ACTIN** |  |  |  |  |  |  | **RHOB** |  |  |  |  | Goulidaki et al., 2015 |
| **TUBULIN** |  |  |  |  |  |  | **RAB5** |  |  |  |  |  |
| **VIMENTIN** |  |  |  |  |  |  | **TGN38** |  |  |  |  |  |
| **ERGIC 53** |  |  |  |  | Seo & Britt, 2006 |  | **GM130** |  |  |  |  | Rebmann et al., 2016 |
| **MAN II** |  |  |  |  |  |  | **GOLGIN245** |  |  |  |  |  |
| **GalT (TGN)** |  |  |  |  |  |  | **GRASP65** |  |  |  |  |  |
| **Golgin 97** |  |  |  |  | Das & Pellett, 2007 |  | **MITOCHONDRIA** |  |  |  |  | Beltran et al., 2016 |
| **EEA1** |  |  |  |  |  |  | **LAMP1** |  |  |  |  |  |
| **TGN38** |  |  |  |  |  |  | **STX5** |  |  |  |  | Cruz et al., 2017 |
| **GM130** |  |  |  |  |  |  | **GM130** |  |  |  |  |  |
| **BiP/GRP78** |  |  |  |  |  |  | **P230** |  |  |  |  |  |
| **GRP78** |  |  |  |  | Das et al., 2007 |  | **TGN46** |  |  |  |  | Pavelin et al., 2017) |
| **GM130** |  |  |  |  |  |  | **EEA1** |  |  |  |  |  |
| **MAN II** |  |  |  |  |  |  | **GM130** |  |  |  |  |  |
| **P230 (TGN** |  |  |  |  |  |  | **CLATHRIN** |  |  |  |  | Archer et al., 2017 |
| **EEA1** |  |  |  |  |  |  | **TGN46** |  |  |  |  | Dietz et al., 2018 |
| **RAB5** |  |  |  |  |  |  | **TfR** |  |  |  |  |  |
| **CHMP1** |  |  |  |  | Tandon et al., 2009 |  | **TGN46** |  |  |  |  | Procter et al., 2018 |
| **VPS4A** |  |  |  |  |  |  | **CDK5RAP2** |  |  |  |  |  |
| **RAB11-FIP4** |  |  |  |  | Krzyzaniak et al., 2009 |  | **LC3** |  |  |  |  | Taisne et al., 2019 |
| **EGFR** |  |  |  |  |  |  | **GM130** |  |  |  |  |  |
| **TfR** |  |  |  |  |  |  | **EEA1** |  |  |  |  |  |
| **CATHEPSIN D** |  |  |  |  |  |  | **GABARAPL1** |  |  |  |  |  |
| **CD63** |  |  |  |  |  |  | **GAT16** |  |  |  |  |  |
| **GIANTIN** |  |  |  |  | Cepeda et al., 2010 |  | **P62** |  |  |  |  |  |
| **TGN46** |  |  |  |  |  |  | **GM130** |  |  |  |  | Turner et al., 2020 |
| **EEA1** |  |  |  |  |  |  | **VAMP3** |  |  |  |  |  |
| **HRS** |  |  |  |  |  |  | **CD63** |  |  |  |  | Hashimoto et al., 2020 |
| **ANNEXIN1** |  |  |  |  |  |  | **IGF2R** |  |  |  |  |  |
| **TfR** |  |  |  |  |  |  | **WDR5** |  |  |  |  | Yang et al., 2022 |
| **CD63** |  |  |  |  |  |  | **EEA1** |  |  |  |  |  |
| **LAMP1** |  |  |  |  |  |  | **CLATHRIN** |  |  |  |  | Momtaz et al., 2021 |
| **CD-M6PR** |  |  |  |  |  |  | **CD63** |  |  |  |  |  |
| **CI-M6PR** |  |  |  |  |  |  | **LBPA** |  |  |  |  |  |
| **RAB27A** |  |  |  |  | Fraile-Ramos et al., 2010 |  | **EEA1** |  |  |  |  |  |
| **CD63** |  |  |  |  | Schauflinger et al., 2011 |  | **RAB5** |  |  |  |  |  |
| **RAB6** |  |  |  |  | Indran & Britt, 2011 |  | **ALIX** |  |  |  |  |  |
| **RAB9** |  |  |  |  |  |  | **RAB7** |  |  |  |  |  |
| **BICD1** |  |  |  |  |  |  | **P230** |  |  |  |  |  |
| **STX3** |  |  |  |  | Cepeda & Fraile-Ramos, 2011 |  | **RAB6** |  |  |  |  |  |
| **BiP** |  |  |  |  | Das & Pellett, 2011 |  | **RAB11** |  |  |  |  |  |
| **GM130** |  |  |  |  |  |  | **RAB14** |  |  |  |  |  |
| **MANN II** |  |  |  |  |  |  | **AP1** |  |  |  |  |  |
| **P230 (TGN)** |  |  |  |  |  |  | **AP2** |  |  |  |  |  |
| **EEA1** |  |  |  |  |  |  | **AP3** |  |  |  |  |  |
| **TfR** |  |  |  |  |  |  |  |  |  |  |  |  |
| **RAB11** |  |  |  |  |  |  |  |  |  |  |  |  |
| **CHMP1A** |  |  |  |  |  |  | Color codes for localization | | | |  |  |
| **VPS4** |  |  |  |  |  |  |  | iAC | oAc | Out |  |  |
| **CD63** |  |  |  |  |  |  | Primary |  |  |  |  |  |
| **LAMP1** |  |  |  |  |  |  | Secondary |  |  |  |  |  |

# Supplementary references

Archer, M. A., Brechtel, T. M., Davis, L. E., Parmar, R. C., Hasan, M. H., & Tandon, R. (2017). Inhibition of endocytic pathways impacts cytomegalovirus maturation. *Scientific Reports*, *7*(April), 1–12. https://doi.org/10.1038/srep46069

Beltran, P. M. J., Mathias, R. A., Cristea, I. M., Beltran, P. M. J., Mathias, R. A., & Cristea, I. M. (2016). A Portrait of the Human Organelle Proteome In Space and Time during Cytomegalovirus Infection. *Cell Systems*, *3*, 361–373. https://doi.org/10.1016/j.cels.2016.08.012

Cepeda, V., Esteban, M., & Fraile-Ramos, A. (2010). Human cytomegalovirus final envelopment on membranes containing both trans-Golgi network and endosomal markers. *Cellular Microbiology*, *12*(3), 386–404. https://doi.org/10.1111/j.1462-5822.2009.01405.x

Cepeda, V., & Fraile-Ramos, A. (2011). A role for the SNARE protein syntaxin 3 in human cytomegalovirus morphogenesis. *Cellular Microbiology*, *13*(6), 846–858. https://doi.org/10.1111/j.1462-5822.2011.01583.x

Cruz, L., Streck, N. T., Ferguson, K., Desai, T., Desai, D. H., Amin, S. G., & Buchkovich, N. J. (2017). Potent Inhibition of Human Cytomegalovirus by Modulation of Cellular SNARE Syntaxin 5. *Journal of Virology*, *91*(1). https://doi.org/10.1128/JVI.01637-16/ASSET/1F1FAD33-81EA-4615-A6EE-177172ADD771/ASSETS/GRAPHIC/ZJV9991822110007.JPEG

Das, S., & Pellett, P. E. (2007). Members of the HCMV US12 family of predicted heptaspanning membrane proteins have unique intracellular distributions, including association with the cytoplasmic virion assembly complex. *Virology*, *361*(2), 263–273. https://doi.org/10.1016/j.virol.2006.11.019

Das, S., & Pellett, P. E. (2011). Spatial Relationships between Markers for Secretory and Endosomal Machinery in Human Cytomegalovirus-Infected Cells versus Those in Uninfected Cells. *Journal of Virology*, *85*(12), 5864–5879. https://doi.org/10.1128/JVI.00155-11/SUPPL_FILE/VIDEO_3DE_1_MBPS.MOV

Das, S., Vasanji, A., & Pellett, P. E. (2007). Three-Dimensional Structure of the Human Cytomegalovirus Cytoplasmic Virion Assembly Complex Includes a Reoriented Secretory Apparatus. *Journal of Virology*, *81*(21), 11861–11869. https://doi.org/10.1128/jvi.01077-07

Dietz, A. N., Villinger, C., Becker, S., Frick, M., & von Einem, J. (2018). A Tyrosine-Based Trafficking Motif of the Tegument Protein pUL71 Is Crucial for Human Cytomegalovirus Secondary Envelopment. *Journal of Virology*, *92*(1). https://doi.org/10.1128/jvi.00907-17

Fraile-Ramos, A., Cepeda, V., Elstak, E., & van der Sluijs, P. (2010). Rab27a is required for human cytomegalovirus assembly. *PLoS ONE*, *5*(12), 1–8. https://doi.org/10.1371/journal.pone.0015318

Goulidaki, N., Alarifi, S., Alkahtani, S. H., Al-Qahtani, A., Spandidos, D. A., Stournaras, C., & Sourvinos, G. (2015). RhoB is a component of the human cytomegalovirus assembly complex and is required for efficient viral production. *Cell Cycle*, *14*(17), 2748–2763. https://doi.org/10.1080/15384101.2015.1066535

Hashimoto, Y., Sheng, X., Murray-Nerger, L. A., & Cristea, I. M. (2020). Temporal dynamics of protein complex formation and dissociation during human cytomegalovirus infection. *Nature Communications*, *11*(1). https://doi.org/10.1038/S41467-020-14586-5

Hook, L. M., Grey, F., Grabski, R., Tirabassi, R., Doyle, T., Hancock, M., Landais, I., Jeng, S., McWeeney, S., Britt, W., & Nelson, J. A. (2014). Cytomegalovirus miRNAs target secretory pathway genes to facilitate formation of the virion assembly compartment and reduce cytokine secretion. *Cell Host and Microbe*, *15*(3), 363–373. https://doi.org/10.1016/j.chom.2014.02.004

Indran, S. v., & Britt, W. J. (2011). A Role for the Small GTPase Rab6 in Assembly of Human Cytomegalovirus. *Journal of Virology*, *85*(10), 5213–5219. https://doi.org/10.1128/jvi.02605-10

Karleuša, L., Lučin, H. M., Zagorac, G. B., & Lučin, P. (2020). Cytoplasmic virion assembly compartment of betaherpesviruses. *Periodicum Biologorum*, *121–122*(3–4), 97–106. https://doi.org/10.18054/pb.v121-122i3-4.10757

Karleuša, L., Mahmutefendić, H., Tomaš, M. I., Zagorac, G. B., & Lučin, P. (2018). Landmarks of endosomal remodeling in the early phase of cytomegalovirus infection. *Virology*, *515*, 108–122. https://doi.org/10.1016/j.virol.2017.12.001

Krzyzaniak, M. A., Mach, M., & Britt, W. J. (2009). HCMV-encoded glycoprotein M (UL100) interacts with rab11 effector protein FIP4. *Traffic*, *10*(10), 1439–1457. https://doi.org/10.1111/j.1600-0854.2009.00967.x

Lacaze, P., Forster, T., Ross, A., Kerr, L. E., Salvo-Chirnside, E., Lisnic, V. J., Lopez-Campos, G. H., Garcia-Ramirez, J. J., Messerle, M., Trgovcich, J., Angulo, A., & Ghazal, P. (2011). Temporal Profiling of the Coding and Noncoding Murine Cytomegalovirus Transcriptomes. *Journal of Virology*, *85*(12), 6065–6076. https://doi.org/10.1128/jvi.02341-10

Liu, S. T. H., Sharon-Friling, R., Ivanova, P., Milne, S. B., Myers, D. S., Rabinowitz, J. D., Brown, H. A., & Shenk, T. (2011). Synaptic vesicle-like lipidome of human cytomegalovirus virions reveals a role for SNARE machinery in virion egress. *Proceedings of the National Academy of Sciences of the United States of America*, *108*(31), 12869–12874. https://doi.org/10.1073/pnas.1109796108

Lučin, P., Jug Vučko, N., Karleuša, L., Mahmutefendić Lučin, H., Blagojević Zagorac, G., Lisnić, B., Pavišić, V., Marcelić, M., Grabušić, K., Brizić, I., & Lukanović Jurić, S. (2020). Cytomegalovirus generates assembly compartment in the early phase of infection by perturbation of host-cell factors recruitment at the early endosome/endosomal recycling compartment/trans-Golgi interface. *Frontiers in Cell and Developmental Biology*, *8*, 914. https://doi.org/10.3389/fcell.2020.563607

Mahmutefendić Lučin, H., Lukanović Jurić, S., Marcelić, M., Štimac, I., Viduka, I., Blagojević Zagorac, G., Lisnić, B., Ruzsics, Z., & Lučin, P. (2023). Membraneless Compartmentalization of Nuclear Assembly Sites during Murine Cytomegalovirus Infection. *Viruses*, *15*(3), 766. https://doi.org/10.3390/V15030766/S1

Marcelić, M., Mahmutefendić Lučin, H., Jurak Begonja, A., Blagojević Zagorac, G., Juranić Lisnić, V., & Lučin, P. (2021). Endosomal phosphatidylinositol-3-phosphate-associated functions are dispensable for establishment of the cytomegalovirus pre-assembly compartment but essential for the virus growth. *Life*, *11*(8), 859. https://doi.org/10.3390/life11080859

Marcinowski, L., Lidschreiber, M., Windhager, L., Rieder, M., Bosse, J. B., Rädle, B., Bonfert, T., Györy, I., de Graaf, M., da Costa, O. P., Rosenstiel, P., Friedel, C. C., Zimmer, R., Ruzsics, Z., & Dölken, L. (2012). Real-time Transcriptional Profiling of Cellular and Viral Gene Expression during Lytic Cytomegalovirus Infection. *PLoS Pathogens*, *8*(9), 6–10. https://doi.org/10.1371/journal.ppat.1002908

Momtaz, S., Molina, B., Mlera, L., Goodrum, F., & Wilson, J. M. (2021). Cell Type-Specific Biogenesis of Novel Vesicles Containing Viral Products in Human Cytomegalovirus Infection. *Journal of Virology*, *95*(11). https://doi.org/10.1128/jvi.02358-20

Pavelin, J., McCormick, D., Chiweshe, S., Ramachandran, S., Lin, Y. T., & Grey, F. (2017). Cellular v-ATPase is required for virion assembly compartment formation in human cytomegalovirus infection. *Open Biology*, *7*(11). https://doi.org/10.6084/m9.figshare.c.3904894

Procter, D. J., Banerjee, A., Nukui, M., Kruse, K., Gaponenko, V., Murphy, E. A., Komarova, Y., & Walsh, D. (2018). The HCMV Assembly Compartment Is a Dynamic Golgi-Derived MTOC that Controls Nuclear Rotation and Virus Spread. *Developmental Cell*, *45*(1), 83-100.e7. https://doi.org/10.1016/j.devcel.2018.03.010

Rebmann GM, R, G., V, S., & WJ, B. (2016). Phosphorylation of Golgi Peripheral Membrane Protein Grasp65 Is an Integral Step in the Formation of the Human Cytomegalovirus Cytoplasmic Assembly Compartment. *MBio*, *7*(5), 1–15. https://doi.org/10.1128/mBio.01554-16

Sanchez, V., & Britt, W. (2022). Human cytomegalovirus egress: Overcoming barriers and co-opting cellular functions. In *Viruses* (Vol. 14, Issue 1, pp. 1–21). https://doi.org/10.3390/v14010015

Sanchez, V., Greis, K. D., Sztul, E., & Britt, W. J. (2000). Accumulation of Virion Tegument and Envelope Proteins in a Stable Cytoplasmic Compartment during Human Cytomegalovirus Replication: Characterization of a Potential Site of Virus Assembly. *Journal of Virology*, *74*(2), 975–986. https://doi.org/10.1128/jvi.74.2.975-986.2000

Schauflinger, M., Fischer, D., Schreiber, A., Chevillotte, M., Walther, P., Mertens, T., & von Einem, J. (2011). The Tegument Protein UL71 of Human Cytomegalovirus Is Involved in Late Envelopment and Affects Multivesicular Bodies. *Journal of Virology*, *85*(8), 3821–3832. https://doi.org/10.1128/jvi.01540-10

Seo, J.-Y., & Britt, W. J. (2006). Sequence Requirements for Localization of Human Cytomegalovirus Tegument Protein pp28 to the Virus Assembly Compartment and for Assembly of Infectious Virus. *Journal of Virology*, *80*(11), 5611–5626. https://doi.org/10.1128/jvi.02630-05

Štimac, I., Vučko, N. J., Zagorac, G. B., Marcelić, M., Lučin, H. M., & Lučin, P. (2021). Dynamin inhibitors prevent the establishment of the cytomegalovirus assembly compartment in the early phase of infection. *Life*, *11*(9). https://doi.org/10.3390/life11090876

Taisne, C., Lussignol, M., Hernandez, E., Moris, A., Mouna, L., & Esclatine, A. (2019). Human cytomegalovirus hijacks the autophagic machinery and LC3 homologs in order to optimize cytoplasmic envelopment of mature infectious particles. *Scientific Reports*, *9*(1), 1–13. https://doi.org/10.1038/s41598-019-41029-z

Tandon, R., AuCoin, D. P., & Mocarski, E. S. (2009). Human Cytomegalovirus Exploits ESCRT Machinery in the Process of Virion Maturation. *Journal of Virology*, *83*(20), 10797–10807. https://doi.org/10.1128/jvi.01093-09

Tomaš, M. I., Kučić, N., Mahmutefendić, H., Blagojević, G., & Lučin, P. (2010). Murine cytomegalovirus perturbs endosomal trafficking of major histocompatibility complex class I molecules in the early phase of infection. *Journal of Virology*, *84*(21), 1101–1112. https://doi.org/10.1128/JVI.00988-10

Turner, D. L., Korneev, D. V., Purdy, J. G., de Marco, A., & Mathias, R. A. (2020). The host exosome pathway underpins biogenesis of the human cytomegalovirus virion. *ELife*, *9*, 1–29. https://doi.org/10.7554/ELIFE.58288

Yang, B., Yao, Y., Cheng, H., Wang, X.-Z., Zhou, Y., Huang, S.-N., Ma, X., Yang, H., Wu, J., Jiang, X., Cheng, S., Sun, J.-Y., Zeng, W.-B., Chen, J., Zhang, F.-K., Shen, H.-J., Gu, J.-Y., McVoy, M. A., Britt, W. J., … Luo, M.-H. (2022). Human Cytomegalovirus Hijacks WD Repeat Domain 11 for Virion Assembly Compartment Formation and Virion Morphogenesis. *Journal of Virology*, *96*(5). https://doi.org/10.1128/JVI.01827-21
